# Spatial heterogeneity in risk-adjusted return of spontaneous circulation after out-of-hospital cardiac arrest: the urbanization-adjusted RACA model

**DOI:** 10.1186/s12873-026-01715-4

**Published:** 2026-08-10

**Authors:** Dokyeong Lee, Martin Bender, Eiko Spielmann, Ulrike Grittner, Christof Prugger, Julian Friebel

**Affiliations:** 1 Institute of Public Health, Charité – Universitätsmedizin Berlin, Corporate Member of Freie Universität Berlin and Humboldt-Universität Zu Berlin, Charitéplatz 1, 10117 Berlin, Germany; 2 Emergency Medical Services Department, Berlin Fire and Rescue Service, Voltairestraße 2, 10179 Berlin, Germany; 3 Institute of Biometry and Clinical Epidemiology, Charité – Universitätsmedizin Berlin, corporate member of Freie Universität Berlin and Humboldt-Universität zu Berlin, Charitéplatz 1, 10117 Berlin, Germany; 4 Berlin Institute of Health, Charité – Universitätsmedizin Berlin, Charitéplatz 1, 10117 Berlin, Germany; 5 Clinic for Cardiology, Angiology and Intensive Care Medicine CBF, Deutsches Herzzentrum der Charité, Hindenburgdamm 30, 12203 Berlin, Germany

**Keywords:** Out-of-hospital cardiac arrest, Return of spontaneous circulation, Risk adjustment, Calibration, Degree of urbanisation (DEGURBA), Benchmarking

## Abstract

**Background:**

Registry-based benchmarking of return of spontaneous circulation (ROSC) requires well-calibrated risk adjustment across Emergency Medical Services (EMS) systems. Existing risk-adjustment models, however, do not account for urbanization-related heterogeneity, which may contribute to systematic urbanization-stratified miscalibration, bias cross-system benchmarking, and misdirect quality improvement. We developed and validated an urbanization-adjusted ROSC after cardiac arrest (URACA) by incorporating Eurostat’s Degree of Urbanisation (DEGURBA) into RACA and assessed whether it improves calibration across urbanization levels without compromising discrimination.

**Methods:**

Using 2014–2023 German Resuscitation Registry data, we identified 145,780 adult (≥18 years) out-of-hospital cardiac arrests and randomly split cases into development and validation cohorts. DEGURBA was recoded as metropolises (≥500,000 inhabitants), cities (<500,000), and non-urban areas (all others). We refitted RACA and fitted URACA using logistic regression with multiple imputation for missing values. In validation, we assessed calibration (calibration belts), discrimination (area under receiver operating characteristic curve; AUROC), prediction error (Brier score), and reclassification (continuous net reclassification improvement; cNRI).

**Results:**

Refitted RACA exhibited urbanization-stratified miscalibration, overpredicting in non-urban areas and underpredicting in metropolises and cities. URACA reduced these systematic deviations: in non-urban areas, predicted ROSC closely matched observed ROSC across the full risk range, and in metropolises and cities, the mid-range underprediction was resolved. Discrimination and overall accuracy were unchanged (AUROC 0.735 vs 0.737; Brier 0.202 for both), while reclassification improved (cNRI 9.4%).

**Conclusions:**

URACA mitigates urbanization-stratified calibration bias while preserving discrimination, enhancing the comparability of risk-adjusted ROSC benchmarking across EMS systems.

**Supplementary Information:**

The online version contains supplementary material available at 10.1186/s12873-026-01715-4.

## Introduction

Resuscitation guidelines and International Liaison Committee on Resuscitation consensus statements emphasize a systems-of-care approach to out-of-hospital cardiac arrest (OHCA) and advocate registry-based, risk-adjusted benchmarking to monitor Emergency Medical Services (EMS) system performance and inform quality improvement [1–4]. Return of spontaneous circulation (ROSC) provides a pragmatic endpoint for EMS benchmarking because it is assessed during resuscitation and routinely captured in OHCA registries [3]. Its use for valid benchmarking, however, rests on well-calibrated risk adjustment [5, 6].

Prior studies show systematic OHCA outcome differences across urbanization levels, even after adjustment for patient and prehospital factors [7–9]. This suggests that area-level factors may remain unaccounted for after standard adjustment and, if systematic, may compromise calibration across settings. Spatial miscalibration matters because it can distort risk-adjusted benchmarking of ROSC by conflating contextual differences with EMS performance, biasing inferences about prehospital care quality.

This concern is exemplified by the RACA score (Return of Spontaneous Circulation After Cardiac Arrest), a well-established model for ROSC risk adjustment introduced by Gräsner et al. [10, 11]. Although RACA discriminates well, calibration has varied by urbanization in the original German derivation cohort and in external European validations. Predicted ROSC tends to exceed observed ROSC in non-urban areas, whereas calibration in urban settings is size-dependent, underpredicting in smaller and overpredicting in larger urban centers [11–16]. These patterns suggest that the RACA predictor set may miss area-level structural context relevant to ROSC, undermining its use as a common basis for valid benchmarking.

Attempts to address spatial heterogeneity using area-level deprivation indices have yielded limited improvements [17], possibly because deprivation primarily captures socioeconomic disadvantage and may miss system-level features associated with prehospital care delivery [18]. Beyond deprivation alone, Eurostat’s Degree of Urbanisation (DEGURBA) offers a harmonized European classification based on population size, population density, and geographical contiguity, dimensions that may shape how prehospital systems support the chain of survival at population level [8, 19–24].

Hence, we developed and validated an urbanization-adjusted RACA model (URACA) by adding DEGURBA to RACA and assessed whether it improves calibration across urbanization levels while preserving discrimination for system-level, risk-adjusted ROSC benchmarking.

## Methods

### Study design and data source

This study developed and validated a risk-adjustment model using German Resuscitation Registry (GRR) data, with approval from the GRR scientific advisory board (AZ2025-03) and the Ethics Commission of Charité – Universitätsmedizin Berlin (EA2/025/24). Reporting followed TRIPOD for multivariable prediction model studies.

The GRR receives contributions from 146 EMS agencies serving 46% of Germany’s population [25]. Each registry entry comprises 118 Utstein-aligned variables spanning resuscitation-related and logistical factors, drawn from documentation by both the advanced life support ambulance team and the physician-staffed EMS response unit. Data are submitted as anonymized entries via a web-based platform with quality and plausibility checks [26].

### Study setting and population

We included all OHCA events recorded between 1 January 2014 and 31 December 2023 in German regions participating in the GRR. Regions joining the GRR from 2022 onward were excluded due to potentially unstable early-phase data quality. We further excluded patients aged < 18 years, cases lacking ROSC information, and cases for which DEGURBA could not be assigned based on the available registry postal-code information.

### RACA variable specification

The eight RACA predictor variables were retained with their original coding and reference categories to isolate the contribution of the urbanization adjustment within URACA. The variables and their reference categories were: sex (female), age (<80 years), presumed etiology (cardiac), witnessed status (non-witnessed), location (at home or workplace), initial ECG rhythm (ventricular fibrillation), and bystander cardiopulmonary resuscitation (CPR; no). EMS response time was treated as a continuous variable as in the original RACA. ROSC was defined as a palpable pulse sustained for >20 s. This follows the GRR’s standardized, Utstein-aligned definition and matches the original score [11].

### Urbanization variable specification

Eurostat’s Degree of Urbanisation (DEGURBA), adopted by the United Nations and the European Union, classifies areas into three categories: (1) cities, (2) towns and suburbs, and (3) rural areas [27]. DEGURBA’s “cities” category (defined as areas with ≥50,000 inhabitants and >50% of the population living in urban center clusters) may over-aggregate larger urban areas and thereby mask potential calibration differences at the upper end of the urban spectrum. Accordingly, we defined “metropolises” as DEGURBA cities with ≥500,000 inhabitants, applying the OECD threshold [28] to year-specific national census data [29].

DEGURBA was therefore recoded into three categories: (1) metropolises (DEGURBA cities with ≥500,000 inhabitants); (2) cities (DEGURBA cities below the metropolis threshold); and (3) towns, suburbs, and rural areas (the remaining DEGURBA categories), collectively termed “non-urban areas”. Metropolises were the reference category. In the GRR, location for county-member municipalities (kreisangehörige Städte) is recorded only at the county (Kreis) level, precluding separation of “towns and suburbs” from “rural areas”. We therefore merged these categories into a non-urban stratum compatible with the available registry spatial resolution and EMS service structure.

The 2014, 2018, and 2020 DEGURBA editions were applied to 2014–2016, 2017–2019, and 2020–2023, respectively. With an unchanged classification scheme and fixed thresholds across editions, temporal shifts in urbanization class indicate demographic change rather than methodological artefacts [27]. To preclude time-dependent bias in the metropolis category, we verified that no GRR-participating region crossed 500,000 inhabitants during 2014–2023. OHCAs were classified via spatial intersection of EMS postal-code polygons and DEGURBA boundaries in QGIS 3.36.3.

### Data preprocessing and model development

Prior to multiple imputation, data were randomly partitioned into a development set (80%) and a hold-out validation set (20%), stratified by urbanization level and ROSC to preserve proportions. Predictor missingness ranged from 0% to 40.7% in the development set and from 0% to 40.9% in the hold-out validation set; variable-specific rates are shown in Table S1. Missingness patterns were inspected by urbanization stratum and broadly similar for most predictors. Missing data were assumed missing at random. The urbanization variable and the outcome had no missing data, as cases with missing values were excluded a priori. Five imputations were generated for development using *mice* with all RACA variables [30], urbanization, and ROSC in the predictor matrix. For the validation set, ROSC was excluded to avoid data leakage.

Within each imputed development set, optimism was estimated using five-fold cross-validation, with four folds used for training and one for validation; potential within-region correlation was acknowledged when interpreting these estimates. We fit two single-level logistic regression models designed to support routine benchmarking with a transportable equation: a refitted RACA model, which re-estimated the intercept and coefficients using the original RACA specification, and URACA, which additionally included urbanization level. Final model coefficients were pooled across imputations using Rubin’s rules [30].

### Model validation

The final model was evaluated in the hold-out set across four domains:

- Calibration: alignment between predicted probabilities and observed event rates, assessed based on the slope/intercept (ideal values: 1 and 0, respectively) [31] and graphical evaluation with calibration belts [32]. Calibration belts graphically compare predicted and observed ROSC probabilities across risk ranges, complementing the calibration intercept and slope.
- Discrimination: separation of event and non-event observations, measured by the area under the receiver operating characteristic curve (AUROC; ≥0.70 considered acceptable [33]), compared between models using DeLong’s test, and supplemented by the Integrated Discrimination Improvement (IDI) to quantify enhanced separation of predicted probabilities [34].
- Prediction error: average deviation of predicted probabilities from observed outcomes, evaluated using the Brier score (values closer to 0 indicating lower prediction error [35]).
- Reclassification: comparative assessment of classification improvement over a baseline, quantified by the continuous Net Reclassification Improvement (cNRI) [34].

Parametric estimates were pooled using Rubin’s rules; *p*-values from non-parametric tests were combined with the D₂ chi-square method [36], and 95% confidence intervals (CIs) were derived from 500 bootstrap replications.

Urbanization-stratified calibration of refitted RACA and URACA was evaluated using 80 and 95% calibration belts. Stratification was applied only to the calibration analyses, consistent with our a priori focus on systematic miscalibration across levels.

### Statistical analysis

Descriptive statistics for continuous and categorical variables were derived from complete cases and stratified by urbanization level. Continuous variables were summarized as means with standard deviations (SDs) or medians with interquartile ranges (IQRs) and categorical variables as frequencies and percentages. Group differences were assessed with the Kruskal–Wallis H test for continuous variables and the Chi-square test for categorical variables, with effect sizes reported as eta-squared and Cramér’s V, respectively.

Regression coefficients and odds ratios (ORs) with 95% CIs were presented. The fitted logistic regression models were used to calculate predicted ROSC probabilities for downstream analysis. Performance metrics were reported along with their corresponding 95% CIs. Sensitivity analysis was conducted on the high-quality reference region subset, defined according to GRR reference-region quality criteria: incidence > 30 per 100,000 inhabitants per year; ROSC rate < 80%; RACA calculable in >60% of cases; and documented follow-up in ≥30% of cases.

Statistical analyses were performed using R Version 4.5.0 (R Foundation for Statistical Computing, Vienna, Austria). All packages and their versions are listed in Table S2.

## Results

**Fig. 1:**
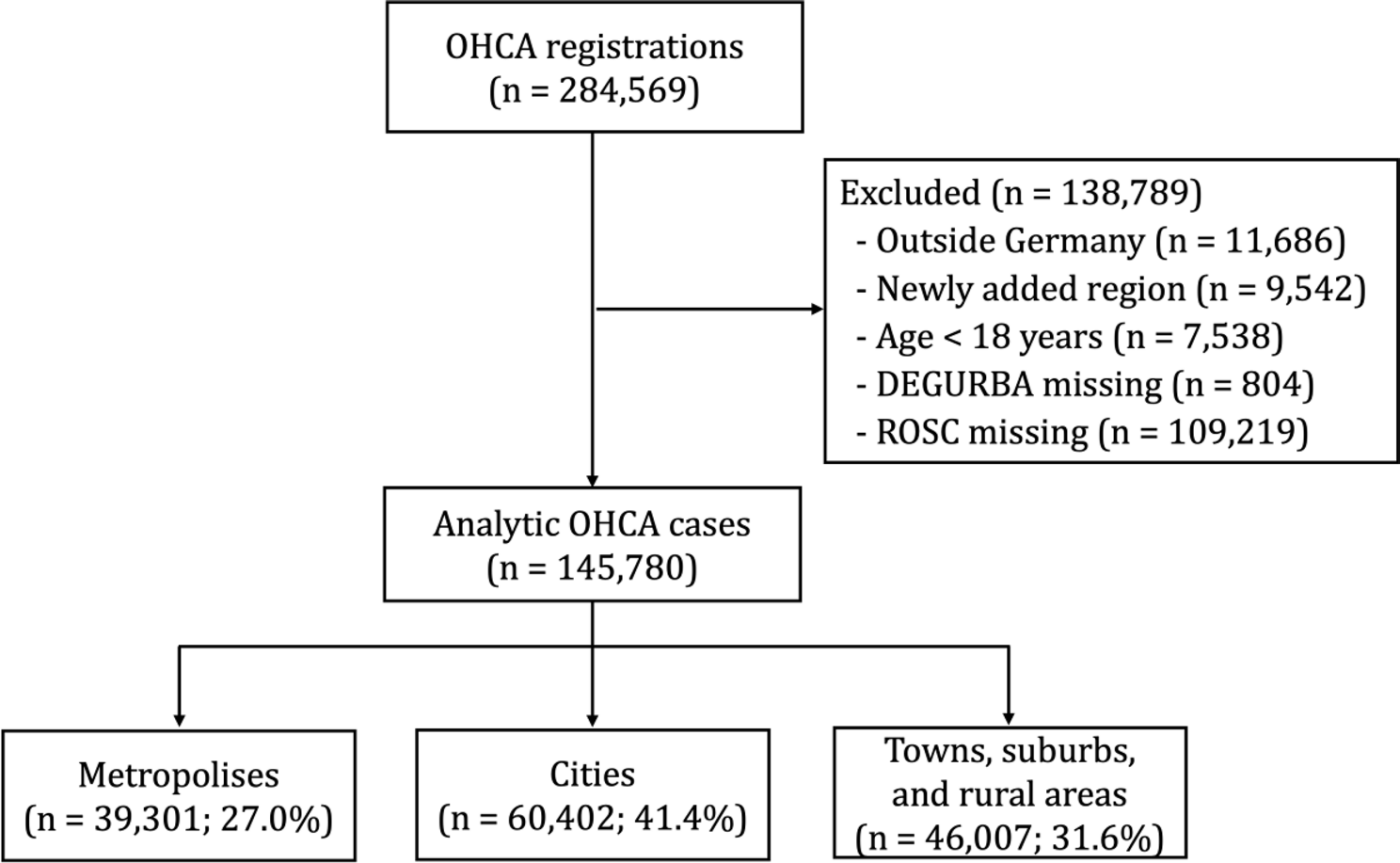
Flowchart of OHCA case selection. OHCA, out-of-hospital cardiac arrest; DEGURBA, degree of urbanisation; ROSC, return of spontaneous circulation

From 1 January 2014 through 31 December 2023, 285,569 OHCAs were registered in the GRR. After prespecified exclusions, 145,780 OHCA cases (51%) remained for analysis (Fig. 1). Baseline characteristics of the development cohort (*n* = 116,626) and the hold-out validation cohort (*n* = 29,154) before multiple imputation are summarized in Table S3. After imputation, the development and validation cohorts remained well balanced: all standardized mean differences were < 0.10 (Cohen’s d for continuous variables; absolute standardized mean difference for categorical variables; Table S4).

### Baseline characteristics by urbanization level

Most OHCAs occurred in cities (41.4%), followed by non-urban areas (31.6%) and metropolises (27.0%) (Fig. 1). Presumed cardiac OHCAs accounted for a larger share in non-urban areas than in metropolises (74.2% vs 68.5%), and the proportion with ventricular fibrillation was likewise slightly higher (24.3% vs 22.6%). OHCAs in non-urban areas occurred at home or the workplace more frequently than in metropolises (69.9% vs 65.6%), and bystander CPR was also more prevalent in non-urban areas (41.8% vs 35.8%). Lay-witnessed arrests were slightly more common in cities and non-urban settings than in metropolises (40.1% and 39.8% vs 38.6%). EMS response times varied modestly across urbanization levels, with medians spanning 8–9 minutes. ROSC rates were 42.1% in non-urban areas, 45.1% in metropolises, and 46.5% in cities. Further details are provided in Table 1.

**Table 1:** Characteristics of RACA variables by Degree of Urbanization^†^

	All (n = 72,972)	Metropolises **(n = 21,263; units = 10)**^**c**^	Cities (n = 29,970; units = 52)	Non-urban areas (n = 21,739; units = 83)	*p*^**d**^	**Effect size**^**e**^
Age (years)^a^	70.1 (24.6)	69.7 (21.1)	70.1 (25.6)	70.6 (26.3)	0.019	η^2^ = 0.000
Sex					0.016	V = 0.011
Female	24,228 (33.3%)	7,236 (34.0%)	9,930 (33.1%)	7,112 (32.8%)		
Male	48,684 (66.7%)	14,027 (66.0%)	20,040 (66.9%)	14,617 (67.2%)		
Presumed Etiology					<0.001	V = 0.043
Cardiac	52,006 (71.4%)	14,567 (68.5%)	21,367 (71.3%)	16,132 (74.2%)		
Trauma	2,195 (3.0%)	651 (3.1%)	911 (3.0%)	633 (2.9%)		
Hypoxia	10,841 (14.9%)	3,639 (17.1%)	4,513 (15.1%)	2,689 (12.4%)		
Intoxication	1,080 (1.5%)	382 (1.8%)	478 (1.6%)	220 (1.0%)		
Other non-cardiac causes	6,790 (9.3%)	2,024 (9.5%)	2,701 (9.0%)	2,065 (9.5%)		
Witnessed by					<0.001	V = 0.014
Laypeople	28,775 (39.4%)	8,216 (38.6%)	12,007 (40.1%)	8,552 (39.3%)		
Professionals	9,983 (13.7%)	2,856 (13.4%)	4,236 (14.1%)	2,891 (13.3%)		
Non-witnessed	34,214 (46.9%)	10,191 (47.9%)	13,727 (45.8%)	10,296 (47.4%)		
Location Type					<0.001	V = 0.033
Home and Workplace	49,122 (67.3%)	13,957 (65.6%)	19,978 (66.7%)	15,187 (69.9%)		
Nursing Home	7,696 (10.5%)	2,448 (11.5%)	3,144 (10.5%)	2,104 (9.7%)		
Doctor’s Office	1,098 (1.5%)	291 (1.4%)	489 (1.6%)	318 (1.5%)		
Public Place	14,000 (19.2%)	4,324 (20.3%)	5,819 (19.4%)	3,857 (17.7%)		
Medical Institution	1,056 (1.4%)	243 (1.1%)	540 (1.8%)	273 (1.3%)		
Initial ECG					<0.001	V = 0.031
VF	17,255 (23.6%)	4,795 (22.6%)	7,174 (23.9%)	5,286 (24.3%)		
PEA	15,499 (21.2%)	4,565 (21.5%)	6,792 (22.7%)	4,142 (19.1%)		
Asystole	40,035 (54.9%)	11,874 (55.8%)	15,913 (53.1%)	12,248 (56.3%)		
Other	183 (0.3%)	29 (0.1%)	91 (0.3%)	63 (0.3%)		
Bystander CPR					<0.001	V = 0.051
Yes	28,993 (39.7%)	7,621 (35.8%)	12,288 (41.0%)	9,084 (41.8%)		
No	43,979 (60.3%)	13,642 (64.2%)	17,682 (59.0%)	12,655 (58.2%)		
EMS response time (min)^b^	9 (6–13)	8 (6–12)	9 (6–13)	9 (6–14)	<0.001	η^2^ = 0.004
ROSC					<0.001	V = 0.037
Yes	32,669 (44.8%)	9,594 (45.1%)	13,925 (46.5%)	9,150‖ (42.1%)		
No	40,303 (55.2%)	11,669 (54.9%)	16,045 (53.5%)	12,589 (57.9%)		

^†^Complete-case analysis

^a^Mean and standard deviation

^b^Median and interquartile range

^c^n denotes the number of individuals; units denote the number of participating geographic regions classified within each urbanization stratum

^d^*p*-values were calculated using rank-based Kruskal–Wallis H tests for continuous variables and chi-square tests for categorical variables; Kruskal–Wallis *p*-values indicate global rank-based distributional differences across groups, rather than differences in group means

^e^η^2^, eta-squared; V, Cramér’s V

ECG, electrocardiogram; PEA, pulseless electrical activity; VF, ventricular fibrillation; CPR, cardiopulmonary resuscitation; EMS, emergency medical service; ROSC, return of spontaneous circulation

### Urbanization-stratified calibration belt analysis

**Fig. 2:**
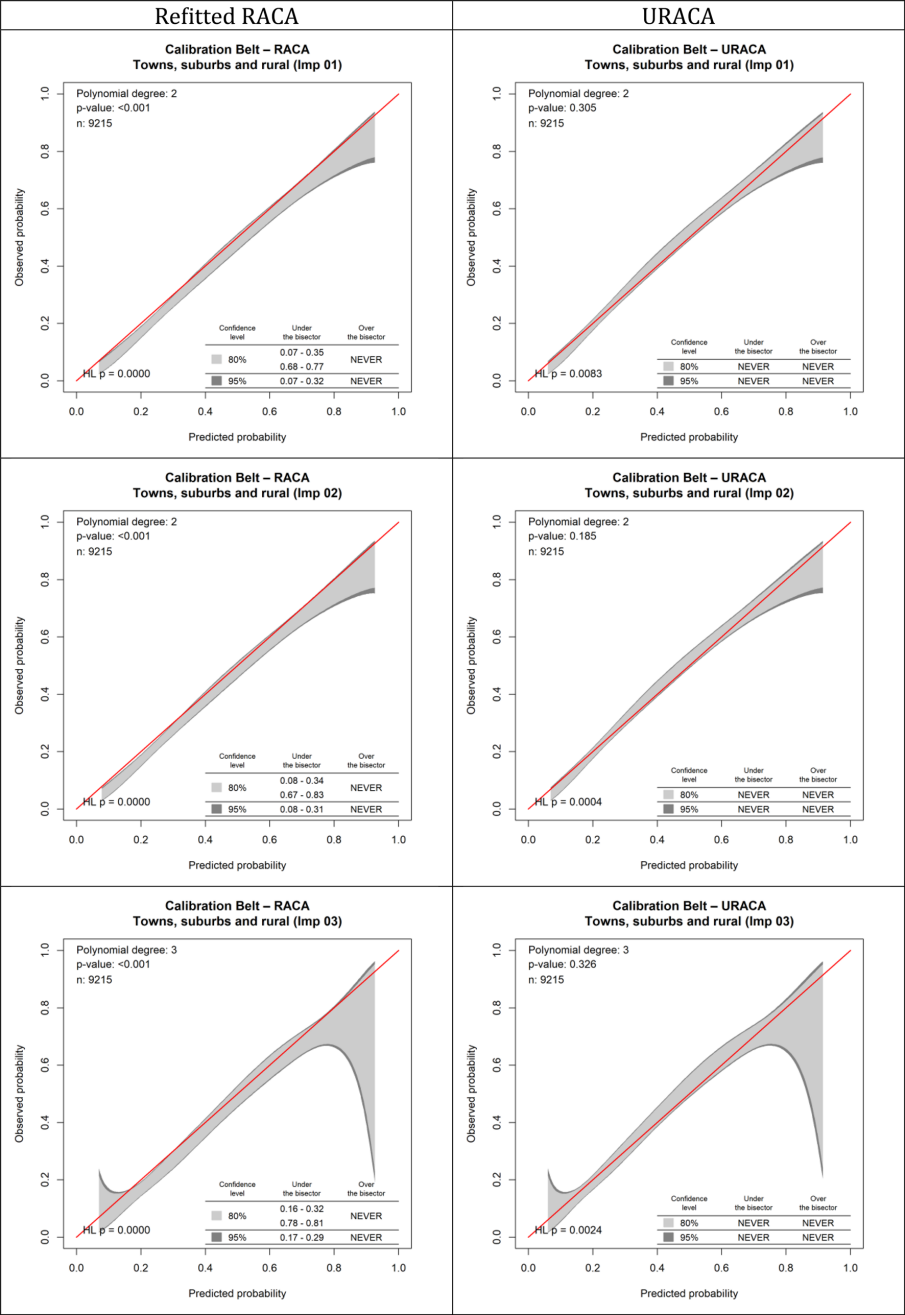

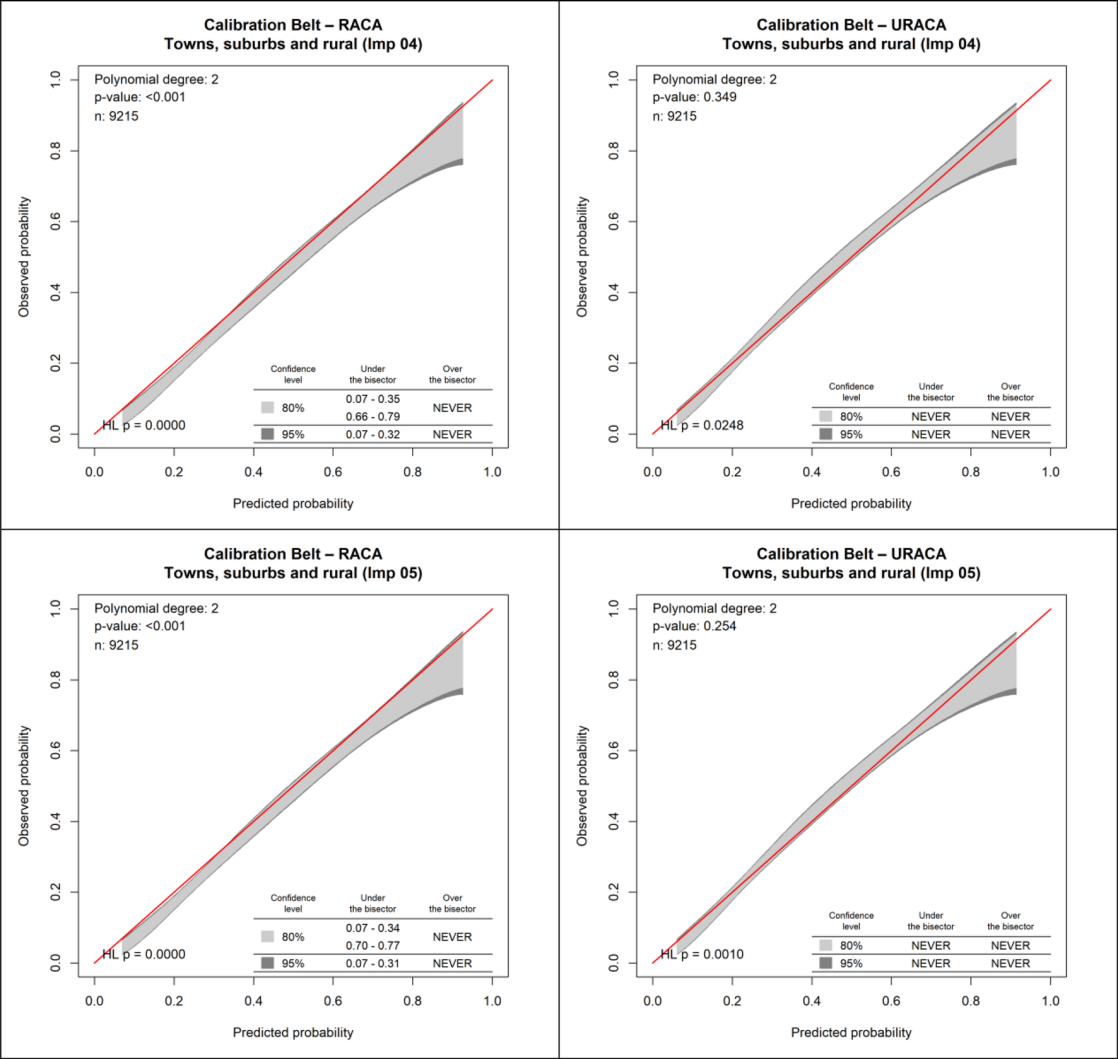
Calibration belts in towns, suburbs and rural areas

Figure 2 shows calibration in non-urban areas, where refitted RACA overestimated ROSC in parts of the predicted-probability range (median *p* < 0.001). After RACA was augmented with the urbanization classification, the calibration belts for URACA closely tracked the 45° line across all five imputations, and the global tests provided no evidence of miscalibration (global *p* = 0.19–0.35; median *p* = 0.31). In metropolises, URACA resolved refitted RACA’s mid-range underestimation at predicted probabilities of 0.31–0.38 (extending up to 0.50 in cities), whereas for predicted probabilities > 0.70, both models continued to overpredict ROSC in both urban settings (Figure S1A–B).

### Overall model performance

Table 2 presents URACA coefficients and odds ratios (95% CIs) and Table 3 summarizes overall model performance. Cox calibration intercepts were close to 0 and slopes were approximately 0.97 for both models. Discrimination was similar (AUROC 0.735 [95% CI 0.729–0.741] for refitted RACA; 0.737 [95% CI 0.731–0.743] for URACA). Brier scores were identical (0.202). URACA improved risk reclassification versus refitted RACA (cNRI 0.094; 95% CI 0.072–0.115; *p* < 0.001), with minimal discrimination gain (IDI 0.001; 95% CI 0.001–0.002) (Table S5). Gains were smaller in the high-quality subset (ΔAUROC 0.0005; cNRI 0.042) (Table S6).

**Table 2:** Coefficients, odds ratios with 95% confidence intervals, and *p*-values from the URACA model

Variable	ß-coefficient	OR (95% CI)	*p*
Sex			
Female	Reference		
Male	−0.27	0.76 (0.74–0.78)	<0.001
Age			
<80 years	Reference		
≥80 years	−0.26	0.77 (0.75–0.80)	<0.001
Etiology			
Cardiac	Reference		
Trauma	−0.67	0.51 (0.47–0.55)	<0.001
Hypoxia	0.55	1.73 (1.66–1.80)	<0.001
Intoxication	0.38	1.46 (1.31–1.63)	<0.001
Other	−0.06	0.95 (0.90–0.99)	0.022
Witnessed by			
None	Reference		
Lay people	0.78	2.18 (2.12–2.25)	<0.001
Professional	0.94	2.57 (2.36–2.80)	<0.001
Location type			
Home and workplace	Reference		
Nursing home	−0.14	0.87 (0.83–0.91)	<0.001
Doctor’s office	0.75	2.12 (1.90–2.37)	<0.001
Public place	0.31	1.37 (1.32–1.42)	<0.001
Medical institution	0.42	1.52 (1.37–1.69)	<0.001
Initial ECG			
VF	Reference		
PEA	−0.88	0.41 (0.40–0.43)	<0.001
Asystole	−1.61	0.20 (0.19–0.21)	<0.001
Other	−0.35	0.71 (0.59–0.84)	<0.001
Bystander CPR			
No	Reference		
Yes	0.00	1.00 (0.97–1.03)	0.919
EMS response time	0.00	1.00 (1.00–1.00)	0.153
Urbanization level			
Metropolises	Reference		
Cities	0.06	1.06 (1.02–1.09)	<0.001
Non-urban areas	−0.18	0.84 (0.81–0.87)	<0.001
Intercept	0.39	1.47 (1.40–1.55)	<0.001

OR, odds ratios; CI, confidence interval; ECG, electrocardiogram; PEA, pulseless electrical activity; VF, ventricular fibrillation; CPR, cardiopulmonary resuscitation; EMS, emergency medical service

**Table 3:** Discrimination and calibration metrics

	Refitted RACA	URACA	*p*
AUROC (95% CI)	0.735 (0.729–0.741)	0.737 (0.731–0.743)	<0.001
Cox calibration			
Intercept	−0.016 (−0.043–0.011)	−0.017 (−0.044–0.010)	
Slope	0.968 (0.938–0.998)	0.965 (0.936–0.995)	
Brier score	0.202 (0.200–0.204)	0.202 (0.199–0.204)	

AUROC, area under the receiver-operating-characteristic curve; CI, confidence interval

## Discussion

To our knowledge, this is the first study using German Resuscitation Registry data (2014–2023) to quantify urbanization-stratified miscalibration in ROSC risk adjustment and examine whether incorporating DEGURBA-defined context improves calibration across urbanization levels. The refitted RACA exhibited urbanization-stratified miscalibration, overpredicting in non-urban areas and underpredicting in metropolises and cities. Adding Eurostat’s DEGURBA-based classification (URACA) removed significant overprediction in non-urban areas and reduced residual calibration bias elsewhere, while preserving discrimination and overall performance.

### Mitigating urbanization-stratified calibration bias

The refitted RACA reproduced the previously reported stratified miscalibration across urbanization levels [12–16], which URACA attenuated. This pattern may reflect system-level structural context at transitions between stages of the chain of survival (e.g. dispatch organization, dispatcher-assisted CPR, and community-response structures), an aspect that may not be adequately captured by case-level factors in RACA. Current American Heart Association and European Resuscitation Council guidelines highlight these interfaces as critical determinants of outcome [1, 2], suggesting the potential clinical relevance of area-level structural context for systems-of-care performance and the interpretability of risk-adjusted benchmarking.

Prior clinical prediction literature indicates that spatial heterogeneity can limit model generalizability and may necessitate setting-specific adjustment, yet it is still uncommon in practice [37, 38]. We build on prior work by showing that including an area-level covariate in ROSC risk adjustment improved agreement between predicted and observed ROSC across urbanization levels, thereby demonstrating the value of accounting for bundled contextual determinants in addressing stratified miscalibration [37, 39].

### Urbanization effects on calibration and discrimination

Incorporating DEGURBA slightly increased the intercept relative to refitted RACA, while leaving the other coefficients largely unchanged, suggesting that urbanization primarily shifts baseline ROSC risk rather than reordering individual risk ranks. This is particularly relevant for benchmarking, because comparability across EMS systems depends primarily on well-calibrated expected outcomes. Thus, the minimal change in AUROC does not undermine URACA’s utility; rather, it is consistent with its calibration-oriented purpose of reducing urbanization-stratified miscalibration. Residual overprediction at higher predicted probabilities in cities and metropolises remains a secondary target for future refinement.

Beyond this benchmarking-oriented role, the limited change in discrimination may partly reflect the broad spatial resolution of DEGURBA. Studies using finer neighborhood-level indicators have reported larger gains in AUROC, whereas our broader urbanization categories may smooth within-area heterogeneity and attenuate contextual signals, a scale-dependent limitation consistent with the modifiable areal unit problem (MAUP) [40, 41]. Future work may therefore explore alternative spatial units and covariates that more directly capture EMS-relevant context, such as EMS catchment areas, dispatch zones, or travel-time surfaces. Nonetheless, DEGURBA offers a pragmatic and standardized approach that can be applied consistently across GRR regions and other DEGURBA-covered European settings.

### Contemporary changes in RACA predictor effects

Compared with the original 1998–2008 RACA cohort, the prognostic weights of EMS response time and bystander CPR were attenuated in our 2014–2023 data. This likely reflects changes in contemporary resuscitation systems rather than reduced clinical importance of time-sensitive prehospital resuscitation interventions. In our cohort, EMS response times were generally clustered below the 10-minute benchmark, which may reduce the marginal effect of additional minutes [42]. In parallel, early chest compressions are now initiated through a broader range of pathways, including dispatcher-assisted CPR and first-responder activation, whereas the original RACA categories do not fully distinguish these routes [43, 44].

Thus, the attenuated weights should be interpreted as evidence of changing prognostic structure rather than diminished value of these interventions, reinforcing the need for periodic updating and contextualization of established benchmarking scores [1, 2].

### Implications for risk-adjusted benchmarking

In URACA, non-urban areas were associated with lower odds of ROSC than metropolises (model-estimated OR = 0.84; 95% CI 0.81–0.87). However, these strata are best interpreted as a pragmatic proxy for area-level structural context, not as a causal explanation for ROSC differences. If unaccounted for, systematic calibration errors persist in ROSC risk adjustment, such that comparable prehospital care quality may appear better or worse depending on the EMS system location. Even small, systematic calibration errors may lead to non-trivial differences in system-level benchmarking outputs because expected ROSC counts are aggregated across large caseloads.

Incorporating the pan-European DEGURBA urbanization classification into the ROSC risk adjustment model reduced the divergence between predicted and observed ROSC across urbanization levels, as shown by stratified calibration belts. This suggests that urbanization-related variation is captured in the expected ROSC probability used for benchmarking rather than being interpreted as performance signals. Risk-adjusted comparisons may thus be less distorted by catchment-area context and more indicative of differences in prehospital care delivery and system performance.

This matters particularly for EMS systems serving DEGURBA-defined non-urban areas, home to about 60% of the EU population. In our study, crude ROSC rates differed by 4.4-percentage-points between cities and non-urban areas. Without adequate risk adjustment, such differences may be interpreted as system performance signals rather than reflection of spatial context.

URACA is not intended to excuse poor performance in non-urban systems; rather, it accounts for contextual constraints to improve the interpretability of adjusted comparisons. Remaining ROSC differences after URACA should be interpreted as benchmarking signals warranting local audit and hypothesis generation, rather than assumptions of inevitable rural underperformance. Candidate domains for review may include structured emergency call triage, dispatcher-assisted CPR, configuration and training intensity of Advanced and Basic Life Support, and advanced airway management previously benchmarked using RACA [45].

URACA may support less biased cross-system benchmarking, thereby strengthening quality improvement efforts in prehospital resuscitation services, with potential downstream benefits for survival with favorable neurological function [1, 2]. For routine benchmarking, external cohorts should establish how URACA affects system-level observed-to-expected ratios and outlier classification across urbanization strata, while broader equity challenges require policy and system interventions beyond risk adjustment.

### Limitations

Several limitations warrant consideration. First, as in registry studies, residual ascertainment or selection bias cannot be excluded and could influence comparisons across urbanization strata. Nevertheless, GRR systematically recorded Utstein-aligned elements with substantial physician-unit coverage, and missingness was broadly comparable across urbanization levels. Second, a single-level logistic model supports a transportable benchmarking equation, but residual region-level confounding may persist; region-specific random intercepts could improve local fit, yet would require site-specific recalibration, undermining comparability across sites. Third, limited spatial granularity necessitated aggregation of ‘towns and suburbs’ and ‘rural areas’ into a single non-urban category, potentially attenuating non-urban heterogeneity and increasing susceptibility to MAUP. Fourth, case-level splitting may yield mildly optimistic validation metrics in the presence of within-region correlation, even with five-fold cross-validation; however, the observed calibration differences are unlikely to be an artefact because both models were evaluated under the same split. Finally, DEGURBA is Europe-specific, but comparable taxonomies exist elsewhere (e.g., the USDA Rural–Urban Continuum Codes).

## Conclusion

URACA mitigates urbanization-stratified calibration bias while preserving discrimination, suggesting that incorporation of urbanization may improve the comparability of risk-adjusted ROSC benchmarking across EMS systems.

## Electronic supplementary material

Below is the link to the electronic supplementary material.

Supplementary material 1

## Data Availability

The data used in this study are not publicly available because of legal and data protection restrictions. Access is governed by the German Resuscitation Registry and subject to its policies and applicable regulations.
