# Supplementary material for: Spatial heterogeneity in risk-adjusted return of spontaneous circulation after out-of-hospital cardiac arrest: the urbanization-adjusted RACA model
Source: BMC Emerg Med. 2026 Aug 10;26:220. doi: 10.1186/s12873-026-01715-4 (PMC13466119; doi:10.1186/s12873-026-01715-4)
Supplement: Supplementary file 1 [file 12873_2026_1715_MOESM1_ESM.docx]

Supplement to: Lee D, Bender M, Spielmann E, Grittner U, Prugger C, Friebel J. Spatial Heterogeneity in Risk-Adjusted Return of Spontaneous Circulation after Out-of-Hospital Cardiac Arrest: The Urbanization-adjusted RACA Model

Contents

Table S1…………………………………………………………………………………………………………………….2

Table S2…………………………………………………………………………………………………………………….3

Table S3…………………………………………………………………………………………………………………….5

Table S4…………………………………………………………………………………………………………………….6

Table S5…………………………………………………………………………………………………………………….7

Table S6…………………………………………………………………………………………………………………….7

Figure S1A.…………………………………………………………………………………………………………………8

Figure S1B.………………………………………………………………………………………………………………10

# Table S1. Variable-specific missingness of candidate predictor variables in the development and validation cohorts

|  | **Development Set (%)** | **Hold-out Validation Set (%)** |
| --- | --- | --- |
| Age | 0.00 | 0.00 |
| Sex | 0.03 | 0.04 |
| Presumed Etiology | 14.63 | 14.43 |
| Witnessed Status | 2.65 | 2.67 |
| Location Type | 5.18 | 5.09 |
| Initial ECG | 3.25 | 3.19 |
| Bystander CPR | 2.20 | 2.16 |
| EMS response time | 40.74 | 40.09 |
| Degree of Urbanization | 0.00 | 0.00 |
| ROSC | 0.00 | 0.00 |

# Table S2. R Session Information

| Purpose | R packages and its versions |
| --- | --- |
| Data import & processing | readxl_1.4.5 |
|  | dplyr_1.1.4 |
|  | tidyr_1.3.1 |
|  | stringr_1.5.1 |
|  | forcats_1.0.0 |
|  | purrr_1.0.4 |
|  | hms_1.1.3 |
|  | lubridate_1.9.4 |
| Missing data exploration & multiple imputation | naniar_1.1.0 |
|  | mice_3.18.0 |
|  | miceadds_3.17-44 |
| Descriptive statistics | dplyr_1.1.4 |
|  | tidyr_1.3.1 |
| Modelling & internal validation | stats (R version 4.5.0) |
|  | caret_7.0-1 |
| Model performance metrics | pROC_1.18.5 |
|  | ResourceSelection_0.3-6 |
|  | givitiR_1.3 |
|  | rmda_1.6 |
| Parallel execution and caching | future_1.49.0 |
|  | future.apply_1.11.3 |
| Graphics & high-resolution export | ggplot2 |
|  | gridExtra_2.3 |
|  | scales_1.4.0 |
|  | ragg_1.4.0 |

# Table S3. Development and Hold-Out Validation Cohorts Before Multiple Imputation^†^

|  | **Development**  **Cohort (n=116,626)** | **Hold-out Validation**  **Cohort (n=29,154)** |
| --- | --- | --- |
| Age (year)^a^ | 72 (60-82) | 72 (60-82) |
| Sex |  |  |
| Female | 40,190 (34.5%) | 9,894 (33.9%) |
| Male | 76,402 (65.5%) | 19,248 (66.0%) |
| NA | 34 (0.03%) | 12 (0.04%) |
| Presumed Etiology | | |
| Cardiac | 69,725 (59.8%) | 17,447 (59.8%) |
| Trauma | 3,410 (2.92%) | 823 (2.82%) |
| Hypoxia | 14,600 (12.5%) | 3,653 (12.5%) |
| Intoxication | 1,468 (1.26%) | 390 (1.34%) |
| Other non-cardiac causes | 10,359 (8.9%) | 2,633 (9.0%) |
| NA | 17,064 (14.6%) | 4,208 (14.4%) |
| Witnessed by | | |
| Laypeople | 47,439 (40.7%) | 11,819 (40.5%) |
| Professionals | 16,326 (14.0%) | 4,101 (14.1%) |
| Non-witnessed | 49,768 (42.7%) | 12,456 (42.7%) |
| NA | 3,093(2.7%) | 778 (2.7%) |
| Location Type |  |  |
| Home and Workplace | 73,968 (63.4%) | 18,535 (63.6%) |
| Nursing Home | 11,991 (10.3%) | 2,948 (10.1%) |
| Doctor’s Office | 1,638 (1.4%) | 395 (1.35%) |
| Public Place | 21,256 (18.2%) | 5,368 (18.4%) |
| Medical Institution | 1,734 (1.5%) | 423 (1.5%) |
| NA | 6,039 (5.2%) | 1,485 (5.1%) |
| Initial ECG | | |
| VF | 23,900 (20.5%) | 6,037 (20.7%) |
| PEA | 22,494 (19.3%) | 5,594 (19.2%) |
| Asystole | 65,811 (56.4%) | 16,418 (56.3%) |
| Other | 629 (0.5%) | 4,30 (0.6%) |
| NA | 3,792 (3.3%) | 929 (3.2%) |
| Bystander CPR |  |  |
| Yes | 43,429 (37.2%) | 10,790 (37.0%) |
| No | 70,627 (60.6%) | 17,734 (60.8%) |
| NA | 2,570 (2.2%) | 630 (2.2%) |
| EMS response time (min)^b,c^ | 9 (6-13) | 9 (6-13) |
| Degree of Urbanization |  |  |
| Metropolis | 31,442 (27.0%) | 7,859 (27.0%) |
| Cities | 48,322 (41.4%) | 12,080 (41.4%) |
| Towns, suburbs, and rural areas | 36,862 (31.6%) | 9,215 (31.6%) |
| ROSC |  |  |
| Yes | 47,701 (40.9%) | 11,924 (40.9%) |
| No | 68,925 (59.1%) | 17,230 (59.1%) |

^†^ ≥1 missing before imputation: n/N=72,808/145,780 (49.9%); complete cases: N=72,972

^a^ Mean and standard deviation

^b^ Median and interquartile range (IQR), with IQR presented as the 25th–75th percentile interval

^c^ Missing: development n=47,513 (40.7%); hold-out validation n=11,923 (40.9%)

CPR, cardiopulmonary resuscitation; ECG, electrocardiogram; PEA, pulseless electrical activity; VF, ventricular fibrillation; EMS, emergency medical service; ROSC, return of spontaneous circulation

# Table S4. Standardized Mean Differences Between the Development and Hold-Out Validation Cohorts After Multiple Imputation

|  | **Mean** | **Standard Deviation** |
| --- | --- | --- |
| Cohens_d for continuous variables |  |  |
| Age (years) | -0.0046 | 0.0039 |
| EMS response time | -0.0032 | 0 |
| ASMD for categorical variables |  |  |
| Sex_Females | 0.0110 | 0.0001 |
| Sex_Males | 0.0110 | 0.0001 |
| Etiology_Cardial | 0.0040 | 0.0023 |
| Etiology_Trauma | 0.0094 | 0.0044 |
| Etiology_Hypoxia | 0.0035 | 0.0017 |
| Etiology_Intoxication | 0.0089 | 0.0042 |
| Etiology_ Other non-cardiac causes | 0.0040 | 0.0015 |
| Witnessed by_Laypeople | 0.0029 | 0.0010 |
| Witnessed by_Professionals | 0.0026 | 0.0010 |
| Witnessed by_None | 0.0011 | 0.0009 |
| Location Type_ Home and Workplace | 0.0024 | 0.0005 |
| Location Type_Nursing Home | 0.0053 | 0.0004 |
| Location Type_Doctor’s Office | 0.0033 | 0.0011 |
| Location Type_Public Place | 0.0033 | 0.0005 |
| Location Type_Medical Institution | 0.0034 | 0.0017 |
| Initial ECG_VF | 0.0058 | 0.0016 |
| Initial ECG_PEA | 0.0033 | 0.0012 |
| Initial ECG_Asystole | 0.0033 | 0.0010 |
| Initial ECG_Other | 0.0080 | 0.0010 |
| Bystander CPR_No | 0.0060 | 0.0007 |
| Bystander CRP_Yes | 0.0060 | 0.0007 |
| Urbanization_Metropolises^†^ | 0.0001 | 0 |
| Urbanization_Cities^†^ | 0.0000 | 0 |
| Urbanization_ Towns, suburbs, and rural areas^†^ | 0.0000 | 0 |

^†^ Urbanization classification complete; excluded from multiple imputation

EMS, emergency medical service; ASMD, absolute standardized mean difference; ECG, electrocardiogram; PEA, pulseless electrical activity; VF, ventricular fibrillation; CPR, cardiopulmonary resuscitation

Table S5. Incremental performance improvement

|  | **Point estimate (95 % CI)** | ***p*** |
| --- | --- | --- |
| ΔAUROC | 0.002 (0.001–0.002) | < .001 |
| Total NRI | 0.094 (0.072–0.115) | < .001 |
| Event NRI | 0.423 (0.407–0.439) |  |
| Non-event NRI | -0.329 (-0.343 – -0.315) |  |
| IDI | 0.001 (0.001–0.002) | < .001 |

AUROC, area under the receiver-operating-characteristic curve; NRI, net reclassification improvement; IDI, integrated discrimination improvement; CI, confidence interval

# Table S6. Incremental Performance Improvement from Registry’s High-Quality Subset^†^

|  | **Point estimate (95 % CI)** | ***p*** |
| --- | --- | --- |
| ΔAUROC | 0.0005 (-0.000–0.001) | .09 |
| Total NRI | 0.042 (0.007–0.078) | .02 |
| Event NRI | 0.134 (0.107–0.161) |  |
| Non-event NRI | -0.092 (-0.115 – -0.069) |  |
| IDI | 0.000 (0.000–0.001) | .05 |

^†^ Within this dataset, 21.4 % of regions are classified as Metropolises, 56.4 % as Cities, and 22.1 % as Towns, suburbs, or rural areas

AUROC, area under the receiver-operating-characteristic curve; NRI, net reclassification improvement; IDI, integrated discrimination improvement; CI, confidence interval

# Figure S1A. Calibration Belts in Metropolises

| Refitted RACA | URACA |
| --- | --- |
|  |  |
|  |  |
|  |  |
|  |  |
|  |  |

# Figure S1B. Calibration Belts in Cities

| Refitted RACA | URACA |
| --- | --- |
|  |  |
|  |  |
|  |  |
|  |  |
|  |  |
